# Dynamic barriers modulate cohesin positioning and genome folding at fixed occupancy

**DOI:** 10.1101/gr.280108.124

**Published:** 2025-08

**Authors:** Hadi Rahmaninejad, Yao Xiao, Maxime M.C. Tortora, Geoffrey Fudenberg

**Affiliations:** Department of Quantitative and Computational Biology, University of Southern California, Los Angeles, California 90089, USA

## Abstract

In mammalian interphase cells, genomes are folded by cohesin loop extrusion limited by directional CTCF barriers. This process enriches cohesin at barriers, isolates neighboring topologically associating domains, and elevates contact frequency between convergent CTCF barriers across the genome. However, recent in vivo measurements present a puzzle: reported CTCF residence times on chromatin are in the range of a few minutes, whereas cohesin lifetimes are much longer. Can the observed features of genome folding result from relatively transient barriers? To address this question, we develop a dynamic barrier model, where CTCF sites switch between bound and unbound states. Using this model, we investigate how barrier dynamics would impact observables for a range of experimental genomic and imaging data sets, including ChIP-seq, Hi-C, and microscopy. We find the interplay of CTCF and cohesin binding timescales influence the strength of each of these features, leaving a signature of barrier dynamics even in the population-averaged snapshots offered by genomic data sets. First, in addition to barrier occupancy, barrier bound times are crucial for instructing features of genome folding. Second, the ratio of boundary to extruder lifetime greatly alters simulated ChIP-seq and simulated Hi-C. Third, large-scale changes in chromosome morphology observed experimentally after increasing extruder lifetime require dynamic barriers. By integrating multiple sources of experimental data, our biophysical model argues that CTCF barrier bound times effectively approach those of cohesin extruder lifetimes. Together, we demonstrate how models that are informed by biophysically measured protein dynamics broaden our understanding of genome folding.

Genome-wide chromosome conformation capture (Hi-C) reveals multiple features of mammalian interphase genome folding at the megabase scale (McCord et al. 2020). First, boundaries partition chromosomes into a series of topologically associating domains (TADs), appearing as squares of enriched contact frequency on the Hi-C maps. Boundaries between adjacent TADs are largely specified by binding sites for the 11-zinc finger protein CTCF (van Ruiten and Rowland 2021). Second, dots in Hi-C contact maps appear as focally enriched regions of pairwise contact frequency and often correspond to convergently oriented CTCF sites. Dots are thought to reflect an increased frequency of physical chromatin loops between convergent CTCF sites (van Ruiten and Rowland 2021). The genomic positions of boundaries and dots anchor all display enrichment for cohesin by ChIP-seq. Acute depletion of CTCF leads to genome-wide loss of TADs and dots in Hi-C contact maps as well as a loss of cohesin ChIP-seq enrichment at CTCF binding sites (Nora et al. 2017). Because altered CTCF binding even at individual positions can lead to dysregulated gene expression and disease (Lupiáñez et al. 2015), it is of great interest to understand how binding specifies folding across the genome.

CTCF is thought to exert its influence on genome folding by acting as a barrier to loop extrusion by the cohesin complex (Fudenberg et al. 2017; van Ruiten and Rowland 2021). During loop extrusion, cohesin binds to chromatin and acts as a motor to progressively create larger chromatin loops until it dissociates. Cohesin accessory proteins modulate parameters of this process; for example, the unloading factor WAPL limits cohesin lifetime and the size of extruded loops. Importantly, CTCF appears to act as a directional barrier to extruding cohesins. The initial understanding of the genome-wide consequences of loop extrusion largely relied on comparisons between simulations and static snapshots obtained from genomic data (Fudenberg et al. 2017; van Ruiten and Rowland 2021).

Observations in living cells, however, argue strongly for a dynamic view of genome organization (Hansen 2020). Protein dynamics of CTCF and cohesin have been quantified with fluorescent recovery after photobleaching (FRAP) and single particle tracking (SPT) in a number of cellular contexts (Fig. 1A; Supplemental Table S1). FRAP data indicates cohesins reside on chromatin with lifetimes of 20–30 min (Gerlich et al. 2006; Tedeschi et al. 2013; Hansen et al. 2017; Morales et al. 2020), whereas CTCF resides with shorter 2- to 11-min lifetimes (Nakahashi et al. 2013; Hansen et al. 2017, 2020; Kieffer-Kwon et al. 2017; Oomen et al. 2019; Soochit et al. 2021; Narducci and Hansen 2025). SPT for CTCF indicated a slightly more rapid 1-min lifetime (Hansen et al. 2017). Tracking DNA, often via integrated arrays of fluorescent reporters, offers a complementary view of dynamics. Importantly, even pairs of genomic loci that appear as salient dots in Hi-C maps are not stably colocalized and make 5- to 30-min contacts (Gabriele et al. 2022; Mach et al. 2022). Single molecule footprinting (SMF), an emerging genomic technology, provides estimates of occupancy of individual CTCF sites. Even strongly bound sites appear to plateau at 70% inferred occupancy (Sönmezer et al. 2021), slightly above an estimated genome-wide average occupancy of 50% (Cattoglio et al. 2019). Each of these technologies argues that dynamic CTCF barriers may enable extruding cohesin motors to bypass individual sites. In vivo, however, no existing approach can concomitantly track multiple genomic positions, cohesin, and CTCF and read out their instantaneous occupancy in real time.

**Figure 1.**
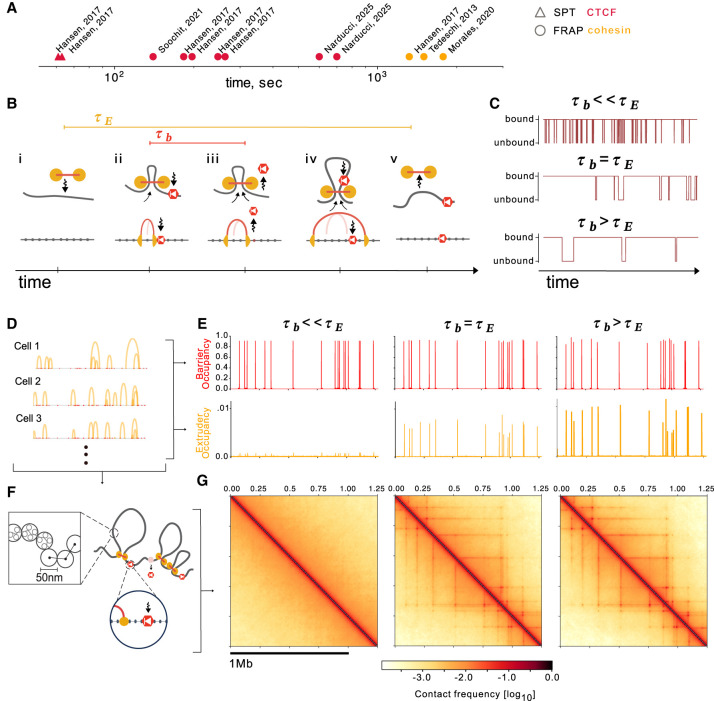
Dynamic barriers show distinct impacts at fixed occupancy. (*A*) Biophysical measurements of residence times for CTCF (red) and cohesin (orange) in mouse cells. Measurements for individual cell lines or acquisition rates shown as separate points. Shapes indicate SPT (triangle) or FRAP (circle). See Supplemental Table S1 for details. (*B*) *Top*: Illustration of CTCF as a dynamic barrier to loop extrusion as a function of time, where CTCF sites are bound with timescale τ_b_ and extruders have lifetime τ_E_. *Bottom*: Lattice implementation. Extruder position at previous time step shown as a light arch. If a barrier becomes unbound (*middle*), an extruder blocked at this site can continue extruding. Note: CTCF can rebind when the barrier is inside of an extruded loop. (*C*) Representative traces for the state of a single barrier (bound or unbound) versus time, for three different regimes of barrier bound time versus extruder lifetime at fixed barrier occupancy. (*D*) Snapshots of extruder positions in different cells generated by the dynamic barriers model for a particular choice of barrier dynamics, where arcs indicate positions connected by the left and right extruder legs. To generate in silico ChIP-seq tracks, extruder positions are recorded for both legs from 500,000 timepoints, representing a population of cells, and averaged. (*E*) In silico ChIP-seq for extruders and barriers across a 1.25-Mb region for three different regimes of extruder versus barrier lifetimes, yet fixed barrier occupancy (0.9). Displayed barrier bound times τ_b_ (4 sec, 1350 sec, 4050 sec) range from much less to much greater than extruder lifetime (τ_e_ = 1350 sec). Barrier ChIP-seq displays equal height peaks, because occupancies are fixed. Extruder ChIP-seq displays strong peaks for long barrier bound times (τ_b_ > τ_e_) and very weak peaks for transient barriers (τ_b_ ≪ τ_e_). Note that variation in extruder peak heights comes from differential spacing between barriers, as all barriers here have identical parameters. (*F*) Extruder positions from 1D lattice simulations are used as input to 3D polymer simulations. Ensembles of 3D conformations are generated to produce contact maps. (*G*) In silico contact maps for the same simulated region and parameters as *E*, binned to 2.5-kb resolution. Barriers with similar (τ_b_ ∼ τ_E_) or longer (τ_b_ > τ_E_) bound times than the extruder lifetime display TADs and dots. In contrast, these patterns vanish with transient barriers (τ_b_ ≪ τ_E_), even at the same occupancy.

Biophysical simulations enable the simultaneous modeling of protein dynamics and genomic observables in silico. Simulations inherently offer independent control over all modeled parameters of the loop extrusion process, including extruder lifetime and barrier bound time. Conformations generated by simulations can be used to extract in silico ChIP-seq, Hi-C, or imaging. Still, existing models either neglected the dynamics of CTCF barriers (Sanborn et al. 2015; Fudenberg et al. 2016; Gabriele et al. 2022) or were not analyzed in terms of residence times as derived from experimental data (Rossini et al. 2022). Thus, the quantitative impacts of CTCF barrier dynamics on ChIP-seq or Hi-C features remain largely unexplored.

To account for the biophysical kinetics of CTCF, we developed a model for loop extrusion with dynamic barriers that stochastically switch between bound and unbound states. We parameterized our simulations with biophysical observations from mouse embryonic stem cells (mESCs) and compared simulation outputs with data from the same cell type. We aimed to unify observations from multiple modalities, including: (i) the fraction of cohesin at CTCF peaks in ChIP-seq; (ii) the strength of insulation and dots in Hi-C contact maps; and (iii) images of whole-chromosome morphology. Together, our approach illustrates how biophysically informed models can be directly confronted with experimental data to sharpen our understanding of genome folding.

## Results

### A model of dynamic CTCF barriers for loop extrusion

To characterize the role of CTCF dynamics for loop extrusion, we extend prior models and explicitly model CTCF occupancy at a set of its cognate sites (Fig. 1B). CTCF sites dynamically switch between an occupied bound state (with bound time τ_b_) and unbound state (with unbound time τ_u_). Importantly, dynamic CTCF sites can maintain the same bound-to-unbound ratio (i.e., have fixed occupancy) while exhibiting distinct dynamics (Fig. 1C). When CTCF sites are occupied, they are unidirectional barriers that prevent loop extruders from passing until they become unbound. This corresponds to experimental evidence preferentially implicating the N-terminus of CTCF for blocking cohesin (Li et al. 2019; Nora et al. 2020; Pugacheva et al. 2020). We simulate loop extrusion dynamics on a 1D lattice at 250-bp resolution with randomly positioned CTCF sites and cohesin lifetime chosen to approximate experimental estimates in mESCs. Reference simulations representing this experimental wild type scenario had an average barrier distance of 75 kb and extruder lifetime of 22 min (Methods). The fine-scale lattice enables modeling CTCF exchanges that are rapid relative to the lifetime of cohesin extruders, while still using the fixed-timestep update scheme used previously (Fudenberg et al. 2016; Nuebler et al. 2018). We note that we do not directly model the search process of individual CTCF molecules but rather account for this with the unbound time (τ_u_) of CTCF sites in our model. Differential search and retention times for CTCF sites across the genome, in principle, allows for a different bound and unbound time at each CTCF site. To characterize key behaviors of the dynamic barriers model across a broad range of parameters, however, we focused on a scenario where all barriers shared the same dynamic timescales. To simulate chromatin, we modeled 2.5 Mb of DNA as a 50-nm fiber with monomers of size 2.5 kb (Methods). Altogether, in our linked 1D extrusion and 3D polymer simulations, loop extruders can sequentially encounter and bypass dynamic barriers with corresponding impacts on the 3D conformation of chromatin (Supplemental Movie).

We generated in silico ChIP-seq and Hi-C from dynamic barrier simulations (Fig. 1D–G). To generate in silico ChIP-seq data (Fig. 1E), we collected the positions of extruders along the 1D lattice across many simulated conformations and computed their average frequency at each position (Fig. 1D). For in silico Hi-C maps (Fig. 1G), we collected conformations from the 3D simulations (Fig. 1F) and recorded all pairs of monomers with spatial distances below a fixed capture radius as contacts (Nuebler et al. 2018; Methods).

At fixed occupancy, in silico ChIP-seq and Hi-C maps are strongly influenced by the CTCF bound time. At high (90%) occupancy, long-lived barriers result in pronounced peaks in ChIP-seq profiles, as well as clearly defined TADs and dots in Hi-C maps, reminiscent of experimental observations (Fig. 1E,G). In contrast, if the barrier bound time is much smaller than the lifetime of extruders (τ_b_ ≪ τ_E_), each of these features largely vanish, similarly to experimental observations for CTCF after degradation (Nora et al. 2017). To determine the range of barrier bound times in vivo, we developed an analytical understanding of barrier dynamics and then compared simulations with multiple quantitative features of genomic data.

### Analytical approach relating loop sizes to barrier dynamics

**Figure 2.**
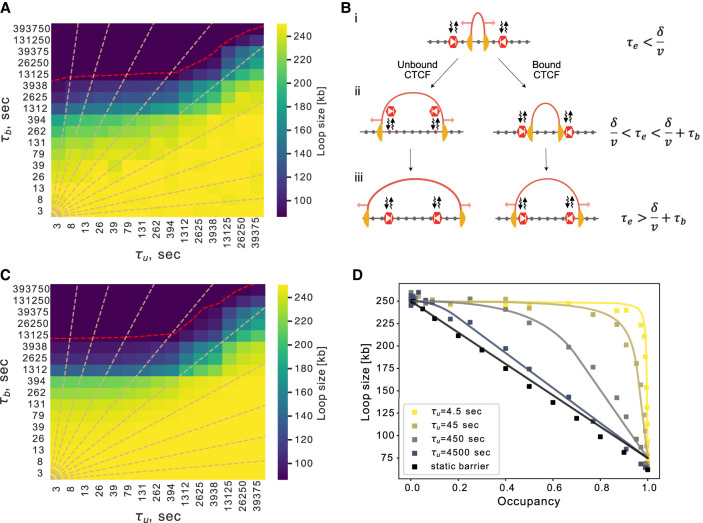
Dynamic barriers lead to distinct regimes for predicted loop size. (*A*) Heat map showing loop sizes as a function of bound (τ_b_) and unbound (τ_u_) times for simulations with a simplified layout of two convergent barriers, as illustrated in *B*. The red dashed line indicates loop size = 87.5 kb, equivalent to the spacing between barriers (δ). Dashed lines indicate constant barrier occupancies between 0.1 and 0.9, spaced by 0.1. (*B*) Illustration of simplified convergent barrier layout and three regimes of extruder lifetime compared to barrier bound times. Red arrows indicate movement of the extruder. At short extruder lifetimes, (i) the extruder has yet to encounter the barrier and moves unimpeded; at intermediate lifetimes, (ii) the extruder becomes stalled if the barrier is occupied, yet proceeds unimpeded if the barrier is unoccupied; at long lifetimes, (iii) the extruder can bypass the barrier position by continued loop enlargement after stalling (*right*), or simply continue unimpeded if the barrier was unoccupied. (*C*) As in *A*, but for the analytical formula for loop size as a function of bound and unbound times. (*D*) Loop size versus occupancy for analytical predictions (lines) versus simulations (dots). Note how curves approach the “static” barrier model at large τ_b_ (darker lines), where loop size is a linearly decreasing function of the occupancy.

To characterize when our simulations would display short versus long barrier bound time behavior, we considered a simplified model that was analytically tractable and quantified how loop sizes depend on barrier dynamics. In the simplified model, a single extruder loads randomly throughout a region between two convergent barriers. This enabled us to quantify how dynamic barriers impact loop sizes without the effect of collisions between extruders, which are known to limit loop growth (Goloborodko et al. 2016). We observed a pronounced decrease of loop size with increasing barrier bound times (Fig. 2A). Barrier unbound time played a role as well. For short unbound times, the decreased loop size appeared at a fixed barrier bound time that was slightly smaller than the extruder lifetime. When unbound time exceeded bound time, however, the sharp decrease of loop sizes occurred at progressively longer bound times. Similar behavior was observed for our original biologically informed layout from Figure 1, although loop sizes were lower due to collisions with other extruders as well as sequential encounters with barriers (Supplemental Fig. S1A).

With the simplified model, three distinct behaviors for loop size emerge (Fig. 2B) depending on extruder lifetime (τ_E_) relative to the barrier distance (δ) divided by extrusion rate (v) and bound time (τ_b_):

1. When extruder lifetime is insufficient to reach the barriers (τ_E_ < δ/v), loop extrusion is unimpeded and loop size is simply proportional to extrusion rate times lifetime (Fig. 2B, i).
2. If extruder lifetime is sufficient to reach the barriers but less than the barrier bound time (δ/v < τ_E_ < δ/v + τ_b_), then the extruder continues unimpeded if it is unoccupied (Fig. 2B, ii, left) but is blocked if it is occupied (Fig. 2B, ii, right).
3. If the lifetime of the extruder exceeds the barrier bound time (δ/v + τ_b_ < τ_E_), the extruder can ultimately bypass the barrier even if its site is occupied (Fig. 2B, iii, right). In this case, the barrier unbinds chromatin before the extruder, and we must account for continued loop enlargement after stalling. As previously, if the barrier was unoccupied, the extruder simply continues unimpeded (Fig. 2B, iii, left).

Considering extrusion behavior in these three regimes allowed us to derive analytical expressions for loop size as a function of extruder lifetime, bound time, unbound time, barrier separation, and extrusion rate (Methods). Our derivation is conceptually similar to previous analytical approaches that characterized the impact of extruder-extruder collisions on loop size (Goloborodko et al. 2016; Banigan and Mirny 2019) and the influence of extrusion on the probability of DNA repair (Yang et al. 2023). However, whereas in those studies barriers were either static or not considered, in our derivation extruder-extruder collisions were neglected.

The equations we derive capture key aspects of barrier dynamics on extruded loop sizes, as evidenced by the similar heat map for loop size as a function of bound and unbound time (Fig. 2A,C). Despite assuming loading at the midpoint between barriers to derive analytical results, clear quantitative agreement is evident in plots of occupancy versus loop size for various unbound times (Fig. 2D). Moreover, equations highlight a sharp change in predicted loop size at extruder lifetime minus barrier distance divided by extrusion rate (τ_E_ – δ/v) at short barrier unbound times (Supplemental Fig. S1B). At long unbound times (τ_u_ ≫ τ_E_), the impact of barriers on loop sizes approaches that of static barriers (Fig. 2D). Analytical expressions reveal the difference between static barriers, where loop size is only sensitive to occupancy, and dynamic barriers, where the bound time plays a central role in determining loop size. Despite these insights, loop sizes remain challenging to measure directly in experiments.

### CTCF dynamics modulate cohesin positioning along the genome

**Figure 3.**
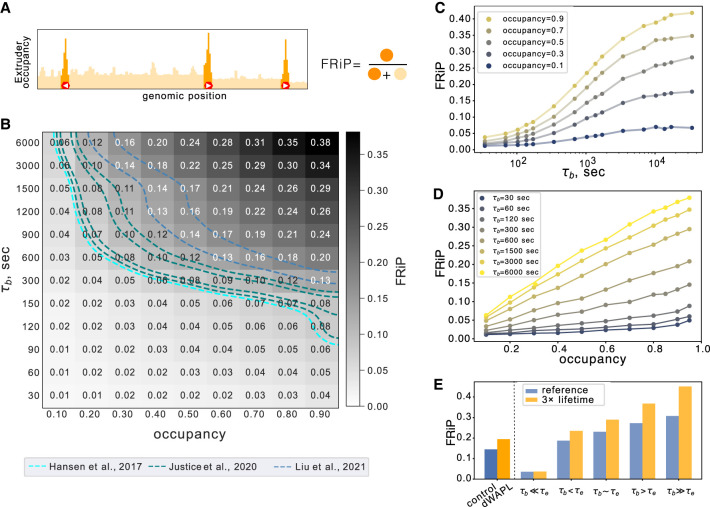
CTCF dynamics modulate cohesin positioning along the genome. (*A*) Illustration of simulated FRiP calculation: after recording extruder leg positions, the fraction that overlap CTCF barrier positions (highlighted in orange) is computed. (*B*) Heat map showing FRiP for CTCF site bound time (τ_b_) versus occupancy. Experimental values (Hansen et al. 2017; Justice et al. 2020; Liu et al. 2021) depicted with dashed lines with values spanning 0.07–0.15. (*C*) FRiP as a function of τ_b_ at fixed occupancy. At small τ_b_, FRiP increases rapidly before reaching a plateau. (*D*) FRiP as a function of occupancy at fixed τ_b_. FRiP increases linearly with occupancy at high τ_b_ but remains low at all occupancies when τ_b_ ≪ τ_E_ (τ_E_ = 1312 sec). (*E*) FRiP before (blue) or after WAPL depletion (yellow) from Liu et al. (2021), alongside simulations with reference (blue) or higher (yellow) lifetime at the indicated barrier bound time (τ_b_).

To characterize the range of CTCF bound times consistent with experimental observations, we considered the predicted impact of dynamic barriers on ChIP-seq, Hi-C, and imaging. Returning to our biologically inspired layout (Fig. 1), we first focused on ChIP-seq due to its high genomic resolution and lower sequencing cost as compared to Hi-C. Simulated ChIP-seq is also less expensive computationally than full 3D polymer simulations, as it is derived from 1D lattice simulations of extruder positions. Experimentally, the enrichment of cohesin at CTCF sites can be quantified as the fraction of cohesin reads in CTCF peaks (FRiP). We similarly quantified our in silico ChIP-seq as the fraction of extruders positioned at barrier locations relative to the total number of extruders (Fig. 3A). Higher values indicate more extruder accumulation at barriers.

To characterize the dynamic barrier model, we quantified FRiP across a range of barrier bound time and occupancies (Fig. 3B; Supplemental Fig. S2A). We found that FRiP generally displayed the opposite trend as loop size as a function of bound time (τ_b_): more potent barriers lead to higher FRiP (Supplemental Fig. S2B). At fixed barrier occupancy, FRiP increased with barrier bound time (Fig. 3C). With increasing bound time, FRiP first displays a rapid increase followed by a plateau. This plateau has equivalent FRiP to a model with static barriers (Supplemental Fig. S2D). Still, FRiP increases with occupancy only at high enough barrier bound time and remains negligible when barriers are transient (Fig. 3D). As for loop sizes, we observed a transition between high and low FRiP regimes when the barrier bound time approaches the extruder lifetime minus the spacing between barriers (i.e., τ_b_ ∼ τ_E_ – δ/v). The strong dependence of FRiP on barrier bound time in simulations aligns with experimental observations that CTCF lifetime better correlates with CTCF-cohesin peak overlap than the CTCF fraction bound across a set of CTCF zinc-finger mutants (Do et al. 2025).

We next considered how other parameters of our model could modulate FRiP. When we plotted FRiP versus occupancy for various unbound times, we found distinct relationships at short and long unbound times (Supplemental Fig. S2C). At short unbound times, the FRiP versus occupancy curve is concave. At long unbound times, the relationship between FRiP and occupancy is roughly linear. Higher extruder lifetime generally led to increased FRiP, similarly to the increase observed experimentally after WAPL depletion (Fig. 2E; Supplemental Fig. S2E). Simulations with higher extruder separation displayed only slightly increased FRiP (Supplemental Fig. S2F). Finally, we tested whether clusters of CTCF sites would yield different FRiP than an isolated site. With a fixed number of bound CTCFs per cluster (i.e., when τ_u_ per barrier was increased proportionally with the number of sites in a cluster), FRiP remained relatively constant (Supplemental Fig. S3A,B).

The strong dependence of FRiP on barrier dynamics in simulations suggested that experimental FRiP could be used to estimate CTCF barrier bound times (see lines for experimental mESC FRiP in Fig. 3B). Assuming an average occupancy of 0.7 (Sönmezer et al. 2021), the dynamic barriers model argues for a bound time between 150 and 900 sec (i.e., 1/10 τ_E_ < τ_b_ < 2/3 τ_E_). Assuming a slightly lower average occupancy gives a slightly higher estimated bound time. Despite being tractable to compute in simulations, we noticed that experimental estimates for FRiP were quite variable. Variability might derive from factors including the choice of antibody and potentially differing levels of background reads between ChIP-seq experiments (Landt et al. 2012). Because background reads are challenging to disentangle from the true signal of cohesin binding across the genome, experimental FRiP only loosely constrains estimates for barrier dynamics. We thus considered whether orthogonal genomic data sets could help constrain estimates for barrier dynamics in vivo.

### Barrier dynamics modify insulation of neighboring TADs

**Figure 4.**
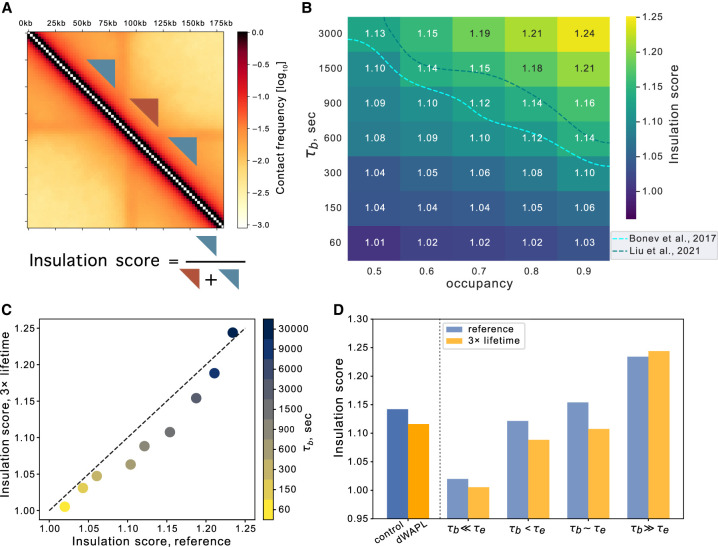
Dynamic barriers modify insulation of neighboring TADs, even at a fixed occupancy rate. (*A*) Illustration of how insulation score is calculated using a window size of 50 kb and equal size triangular areas within (blue) and between (brown) TADs. (*B*) Heat map showing sweeps of insulation score for barrier bound time (τ_b_) versus occupancy, averaged across all simulated barriers. At large τ_b_ and high occupancy, TADs are more isolated. Experimental insulation scores shown as dashed lines (light blue [Bonev et al. 2017] mESC Hi-C; dark blue [Liu et al. 2021] control Hi-C) (also see Supplemental Fig. S4). (*C*) Insulation scores in normal versus threefold increased extruder lifetime conditions for various barrier bound times at occupancy 0.7. Point color reflects bound time. (*D*) Insulation scores from Hi-C before (blue) or after WAPL depletion (yellow) from Liu et al. (2021), alongside simulations with reference (blue) or higher (yellow) lifetime at indicated τ_b_.

To determine the impact of dynamic barriers on 3D genome organization, we quantified patterns in simulated contact maps across a range of barrier bound times. We first quantified the insulation imposed by dynamic barriers on upstream and downstream regions. We defined insulation as the relative frequency of contacts in triangular regions within TADs versus between neighboring TADs (Fig. 4A; Methods). We found a strong dependence of TAD insulation on CTCF dynamics, where TAD insulation increases with both barrier bound time and occupancy (Fig. 4B). This observation aligns with experimental findings where reduced CTCF residence time, caused by ZF8 deletion, weakens insulation between neighboring TADs (Soochit et al. 2021). As for FRiP, insulation varied with both barrier bound times and unbound time, even at fixed occupancy rates. Importantly, high occupancy alone was insufficient to produce strong TADs in simulated Hi-C maps, as sufficient barrier bound times are also required. Comparison with an average experimental insulation score and occupancy suggests barrier bound times approaching that of the extruder lifetime (600–1500 sec).

In simulations with increased extruder lifetimes, we observed lower insulation between TADs for intermediate barrier bound times (Fig. 4C,D; Supplemental Fig. S4A,B). Decreased insulation was observed after experimental WAPL depletion, where cohesin lifetime is thought to increase (Fig. 4D; Supplemental Fig. S4C). Hi-C data thus display a signature of dynamic barriers, as insulation score does not decrease in the static barrier regime (i.e., when τ_b_ ≫ τ_E_) (Fig. 4C,D). The impact of extruder lifetime on insulation contrasts with its effect on FRiP. Whereas FRiP and insulation scores are correlated for fixed extruder parameters and varying barrier bound times, they were often anticorrelated across differing extruder lifetimes (Supplemental Fig. S4D). We note that this presents an instance of Simpson's paradox (Blyth 1972), where a trend observed within individual groups (in this case, extruders with either reference or increased lifetime) reverses when the groups are combined (across bound times). We hypothesize different behavior arises because the insulation score depends on all polymer positions around barriers, whereas ChIP-seq FRiP depends primarily on encounters directly at the barriers.

### Hi-C dot strength versus genomic distance requires dynamic boundaries

**Figure 5.**
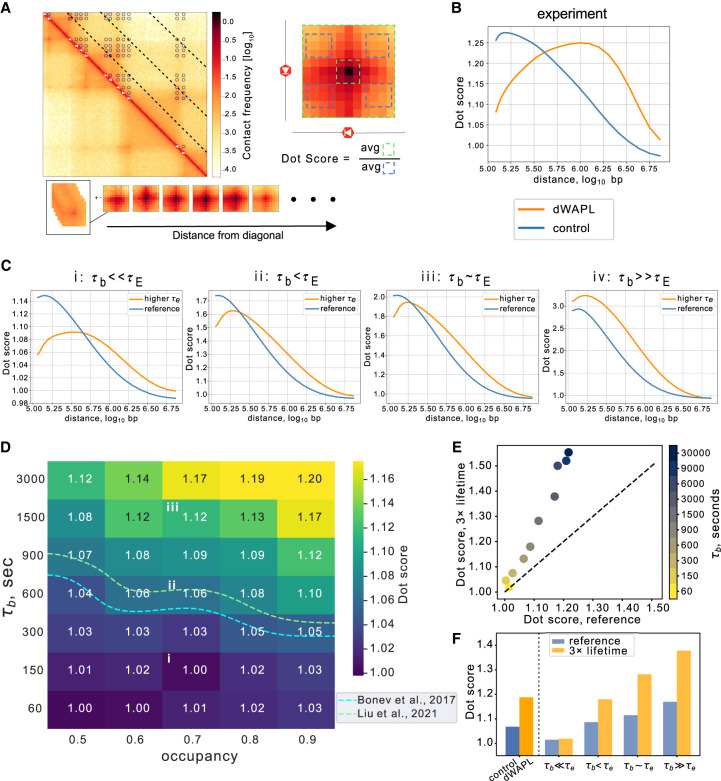
Hi-C dot strength versus distance requires dynamic boundaries. (*A*) Convergent dot scores as a function of genomic distance were calculated between pairs of convergent barriers from contact maps binned to 10-kb resolution. First, genomic distances were binned into 25 logarithmically spaced bins between 100 and 5000 kb (dashed lines overlaid on map). Second, 80-kb snippets centered on barrier pairs in each genomic distance range were collected and averaged (snippet centers shown as dots overlaid on map, averaging shown *below*). Dots scores at each genomic distance were computed as the ratio of contacts in the snippet center relative to four control regions (*right*). (*B*) Convergent dot scores as a function of genomic distance for experimental control (blue) and WAPL depletion data sets (yellow), both from Liu et al. (2021). (*C*) Convergent dot scores as a function of genomic distance for simulations. Plots show extruders with either reference (blue) and higher (orange) lifetimes at three CTCF bound times (i: 150 sec, ii: 600 sec, iii: 1500 sec, iv: 9000 sec). Only simulations with τ_b_ < τ_E_ displayed similar behavior to experiments (ii), where the higher-lifetime curve starts lower and has a peak after the wild-type curve. (*D*) Heat map of simulated distance-averaged dot scores for convergent pairs of barriers over all distances. Note higher scores for larger τ_b_ at fixed occupancies. Dashed lines indicate experimental dot scores for mESCs from Bonev et al. (2017) or control from Liu et al. (2021), and i–iii indicate dot scores computed from curves in *C*. Note that τ_b_ in iv exceeds the displayed range in the heat map. (*E*) Distance-averaged convergent dot score for extruders with reference or higher lifetimes, indicating stronger dots for the latter, particularly at higher τ_b_. (*F*) Distance-averaged convergent dot scores for experimental and simulated data. Experimental dot scores from Liu et al. (2021) increased after WAPL depletion. Simulations for indicated τ_b_ shown for either reference (blue) or increased (yellow) lifetime.

We next investigated how the strength of Hi-C dots depended on simulated CTCF barrier dynamics. As dots correspond to pairs of positions with accumulated extruders, we hypothesized that dot strength might be more closely correlated with FRiP than TAD insulation. We defined dot strength as the frequency of contacts between all possible pairs of convergent barriers relative to their surrounding areas (Fig. 5A; Methods; Flyamer et al. 2020). We used a CTCF barrier-based analysis, because quantifications that rely on calling dots display a bias in computed dot strengths in favor of their reference samples (Supplemental Fig. S5). We note, however, that the less biased barrier-based approach leads to average dot scores substantially lower than the enrichment of the strongest dots. A distinct aspect of dot strength from FRiP or insulation is that it depends on two genomic positions instead of one and is, hence, a function of the genomic distance between dot anchors. We computed the dot strength as a function of genomic distance between convergent CTCF barriers as the ratio of contacts between dot pixels and a local background (Fig. 5B). As in previous studies (e.g., Rao et al. 2014), we compute scores relative to a local background because a global background leads to high scores along any enriched contact stripe or at the edge of any domain. We considered dot strengths across genomic distances between 100 kb and 5 Mb, a range based on the amount of simulated chromatin that enables quantification in simulations with either reference or higher extruder lifetimes. We observed similar trends for dot scores versus distance in both wild-type or control mESC Hi-C (Bonev et al. 2017; Liu et al. 2021) and Micro-C data sets (Hsieh et al. 2020), where scores first increased to a local maximum and then decreased back to baseline levels (Supplemental Fig. S5D). We then compared with the simulated results across a range of barrier lifetimes and fixed occupancy (∼70%) (Fig. 5C; Supplemental Fig. S6C–E). Qualitatively, barriers with very short or very long lifetimes disagreed with experimental curves for dot strength versus distance.

To obtain a single value representing the strength of dots for a particular set of dynamic barrier parameters and quantitatively compare simulations with experiments, we computed averaged dot strengths across a range of genomic distances (Fig. 5D). Similarly to both FRiP and TAD insulation, we observed that dot strength depended strongly on barrier bound time and occupancy, consistent with experimental findings (Fig. 5D; Soochit et al. 2021). Comparison with experimental data suggested a barrier bound time of 300–900 sec, similar to the estimate from insulation scores.

We next considered how modulating extruder lifetime altered dot strength (Supplemental Fig. S6). In simulations with high barrier bound times, increased extruder lifetime enhanced average dot strength across all genomic distances. However, experimentally increasing extruder lifetime via WAPL depletion does not substantially increase the maximal average dot strength (Fig. 5B). Instead, the maximal dot strength in dWAPL shifts to further genomic distances, with weaker dot strengths at moderate (100 kb) genomic distances. In simulations, we found the shift to further genomic distances for higher lifetime extruders can be observed when barriers display more rapid dynamics (Fig. 5C), yet cannot be observed in models with static or very long-lived barriers (Supplemental Fig. S6F). Simulations with dynamic barriers also better reproduced the comparable magnitude of dot strength versus distance curves after WAPL depletion than static or very long-lived barriers (Supplemental Fig. S6C). Despite being calculated from the same simulated Hi-C maps, we observed a different behavior for dot strength versus insulation scores as a function of extruder lifetime. Indeed, for higher extruder lifetimes, we found that dot strength was increased at all barrier bound times (Fig. 5E,F; Supplemental Fig. S6A,B). Collectively, our analyses indicate how dot strength versus distance constitutes a new Hi-C signature of barrier dynamics in vivo.

### Local barrier dynamics can shape whole-chromosome morphology

**Figure 6.**
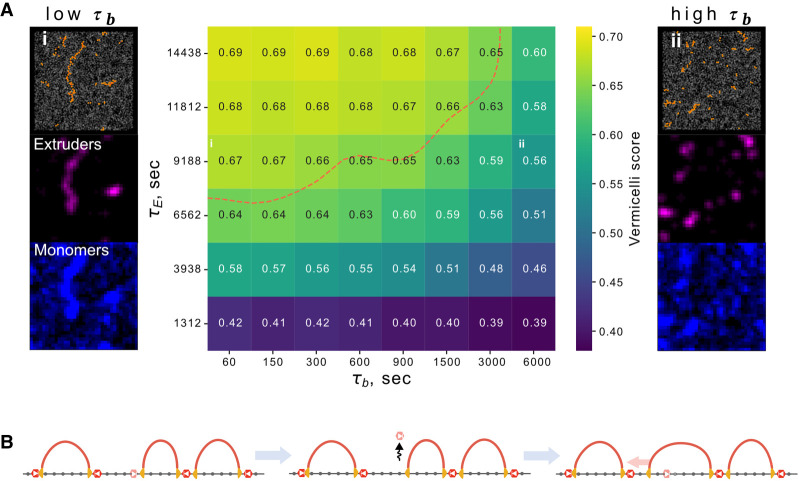
Local barrier dynamics can shape whole-chromosome morphology. (*A*) Heat map indicating vermicelli conformation at various barrier bound times τ_b_ relative to extruder lifetime (τ_E_) at fixed barrier occupancy (0.7). The red dashed line indicates parameter sets displaying prominent vermicelli (scores above 0.65). *Left column*: representative conformation above simulated microscopy for chromatin (blue) and extruders (purple) at sevenfold increased extruder lifetime approximating WAPL depletion and very short barrier bound time (τ_b_ ≪ τ_E_), position in heat map indicated by (i). *Right column*: Similarly, albeit for very long barrier bound time (τ_b_ ≫ τ_E_), with position in heat map indicated by (ii). (*B*) Illustration of how dynamic barriers can have global consequences for vermicelli formation by enabling gap closure between consecutive loops. In this three-step process, an extruder is halted at a barrier next to a gap; the barrier unbinds, allowing extruder bypass; finally, the gap can be closed, irrespective of whether the barrier rebinds.

When cohesin lifetime on chromatin is experimentally increased following WAPL depletion, chromosomes condense and assume a vermicelli-like morphology (Tedeschi et al. 2013). The axes of vermicelli chromatids are enriched for both cohesin and CTCF. Whereas a similar vermicelli morphology can emerge with increased extruder lifetime in simulations, the impact of barrier dynamics on vermicelli formation has not been characterized. If we increased extruder lifetime sevenfold (as estimated experimentally [Wutz et al. 2017]) with transient barriers (τ_b_ << τ_E_), we observed vermicelli formation in simulations (Fig. 6A). At that same extruder lifetime and barrier occupancy, if we increased barrier bound time to that approaching the static regime (τ_b_ >> τ_E_), we no longer observed vermicelli formation. To quantify vermicelli formation, we computed Pearson's correlation between the spatial positions of extruders and chromatin in 3D (Methods; Tortora and Fudenberg 2024). We then assayed simulated vermicelli formation across a range of extruder lifetimes and barrier bound times for fixed barrier occupancy. At reference extruder lifetimes, vermicelli are not present, and there is a low spatial overlap between cohesin and chromatin. At higher extruder lifetimes, a vermicelli morphology is observed. However, if barrier bound times are also high, vermicelli formation is impeded. The experimental observation of vermicelli after an estimated sevenfold increase in lifetime therefore allows us to use simulations to estimate an upper bound on the barrier bound time of 900 sec (∼½τ_E_). As for FRiP, insulation and dot strength, vermicelli formation depended on barrier bound time even at fixed occupancies. How dynamic barriers enable vermicelli formation can be understood by considering the case where two CTCF sites are close together without an extruder in the interval: instead of relying on a low probability event that an extruder is loaded between the barriers, this gap can be closed if an extruder is released after either barrier unbinds (Fig. 6B). In sum, vermicelli formation relies on a delicate balance between barrier dynamics and the extruder lifetime.

In addition to inducing a vermicelli morphology, WAPL depletion also causes grids of dots between more distal CTCF sites to emerge in contact maps (Haarhuis et al. 2017). The dynamic barriers model enables two pathways that could promote the formation of distal dots, either via (i) direct loops that have bypassed intervening barriers, or (ii) stacked loops from collisions between multiple intervening extruders (Supplemental Fig. S7). The latter pathway has been suggested by a combination of imaging, genomic, and perturbation approaches to play roles for organizing multiple genomic loci (Chen et al. 2023; Hafner et al. 2023; Huang et al. 2023). Indeed, increased dot strength for pairs of divergently oriented barriers in dWAPL data, as well as in dynamic barriers simulations with increased lifetime, lends support to the importance of loop stacking (Supplemental Fig. S6E). In our simulations with unidirectional barriers, this arises when higher-lifetime extruders blocked by a convergent barrier can, in turn, act as a barrier to extruders approaching from the alternate direction. We thus considered how the contributions of the two pathways for distal dot enrichment varied specifically for convergent barriers. We quantified how often distal convergent barriers were connected by either a direct loop or stacked loops (with 1–5 intervening barriers) (Supplemental Fig. S7). We found that at fixed barrier occupancy, both direct and stacked loops become more prevalent with increasing barrier bound time and extruder lifetime. For the range of barrier separations considered, the pathway involving direct loops via bypass generally occurred at higher rates. However, the relative frequency of stacked versus direct loops between convergent barriers increases both as a function of the number of intervening barriers and the barrier bound time. We thus hypothesize that loop stacking will be most important at genomic loci decorated by multiple long-lived CTCF barrier sites.

## Discussion

**Figure 7.**
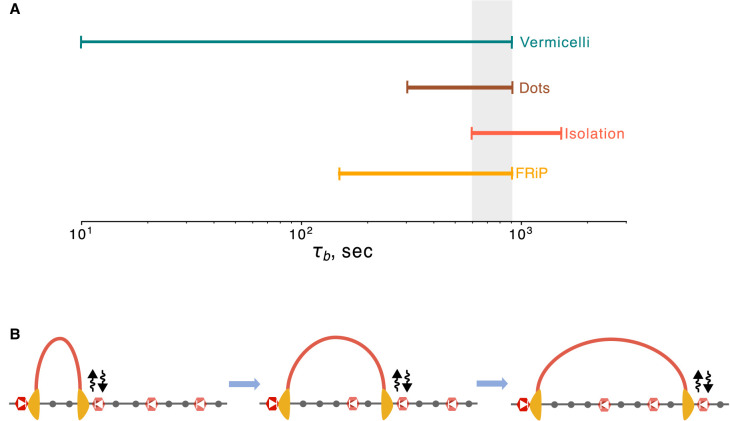
Dynamic barriers are required for agreement with multiple features of experimental data. (*A*) Range of barrier bound times from the dynamic barriers model in agreement with experimental data based on FRiP, insulation score, dot score, and vermicelli formation. Highlighted area (gray) indicates region of agreement across metrics (τ_b_ between 10 and 15 min). (*B*) Consequence of dynamic barrier model: long-lived extruders can sequentially bypass a series of dynamic barriers. This can equalize contact frequencies between a long-lived barrier, acting as an anchor (*left* site, dark red), and multiple downstream dynamic barrier positions (*right* three sites, lighter red).

By characterizing a dynamic CTCF barrier model for interphase loop extrusion, we found that the timescales of barrier exchange modulate observables from multiple modalities across all scales of genome organization. Despite the fact that genomic data are population-averaged snapshots, our quantitative modeling revealed multiple signatures that are consistent with dynamic barriers, yet incompatible with static barriers. Moreover, we find that barriers with identical occupancy can yield distinct predictions for ChIP-seq, TADs, corner dots, and chromosome morphology. Dynamic barriers with bound times of 10–15 min (i.e., between ½ to ⅔ of the extruder lifetime) (Fig. 7A) produced ChIP-seq FRiP, TADs, dots, and vermicelli in good agreement with experimental data.

Using simulations and analytical approaches, we find that the behavior of our dynamic barriers model has three regimes that depend on the ratio of CTCF binding time (τ_b_) to extruder lifetime (τ_E_):

- *Transient barriers* (τ_b_ ≪ τ_E_): extruders easily bypass barriers.
- *Dynamic barriers* (τ_b_ ∼ τ_E_): extruders stall at barriers with occasional release.
- *Quasi-static barriers* (τ_b_ ≫ τ_E_): when a barrier is present, it almost permanently stalls extruders.

We note that our analytical formulae for loop size as a function of barrier dynamics neglect extruder-extruder collisions, which generally limit loop growth (Goloborodko et al. 2016). Still, we observed similar trends in simulations for how dynamic barriers limit loop growth either with or without extruder-extruder collisions (Supplemental Fig. S1A). In the future, it will be valuable to develop analytical approaches that can predict loop size and experimental observables in the combined presence of extruder-barrier collisions and extruder-extruder collisions.

Our simulations predict that barrier bound time is more important than occupancy for determining many features observed in experiments. This arises because barriers require sufficient bound time to block and accumulate extruders. Aligning with our predictions, a higher importance for bound time versus occupancy was recently observed experimentally for cohesin ChIP-seq in a panel of CTCF zinc-finger mutants (Do et al. 2025). Moreover, dynamic barriers simulations with reduced CTCF residence times reduce insulation and dot strengths as seen experimentally via Hi-C for CTCF with a ZF8 deletion (Soochit et al. 2021). Finally, simulations could provide an additional explanation for why many CTCF ChIP-seq peaks (20%–50%) do not display corresponding cohesin peaks, beyond potential issues with antibody quality (Pugacheva et al. 2020). Indeed, even if a given CTCF peak has substantial occupancy, it might fail to impede and accumulate extruding cohesins if the bound time of the site is too short.

Dynamic barriers are additionally required to explain three features of genome folding after WAPL depletion, where the lifetime of cohesin loop extruders is thought to greatly increase (Tedeschi et al. 2013; Haarhuis et al. 2017; Wutz et al. 2017). First, in dWAPL cells, the maximal dot strength between convergent CTCF sites shifts to further genomic distances. This can be recapitulated with dynamic barriers, yet not with static barriers. Second, insulation decreases after dWAPL, which again cannot be recapitulated by static barriers. Third, dWAPL chromosomes adopt a vermicelli morphology. When simulated extruder lifetime is increased, dynamic barriers allow bypass and vermicelli formation in addition to promoting distal dots between divergent barriers via stacking. In contrast, static barriers disrupt simulated vermicelli formation even for very high lifetime extruders. A consequence of dynamic barriers is that longer-lived extruders could potentially bypass multiple barriers sequentially, equalizing contact frequencies across a large genomic region (Fig. 7B). We hypothesize that this feature of dynamic barriers is harnessed to diversify promoter choice at the protocadherin locus to specify neuronal identity (Kiefer et al. 2023, 2024) and diversify V(D)J recombination to specify antibody repertoires (Zhang et al. 2019; Hill et al. 2020; Kiefer et al. 2023).

Whereas FRAP and SPT are insightful for their ability to measure CTCF bound times in vivo, both techniques extract average times across the genome. Across the genome, however, individual sites appear to have a range of biophysical properties (Luan et al. 2021). Given the central role of barrier bound time we reveal, progress in modeling interphase genome folding will require experimental approaches to measure site-specific CTCF residence times, perhaps by adapting methodology from emerging microscopy approaches (Pomp et al. 2024). As such experiments become available, it will be interesting to consider simulations with both site-specific bound times and occupancy (Ramani et al. 2019; Sönmezer et al. 2021), as well as site-specific unbound times. Site-specific unbound times could arise from factors ranging from facilitated diffusion and clustering of sites (Zabet and Adryan 2013) to CTCF-RNA associations (Hansen et al. 2020) that alter CTCF search kinetics.

In addition to varying across the genome, biophysical dynamics of cohesin and CTCF likely vary across cell types and organisms. The range of cohesin lifetimes reported in vivo for different cell types likely reflects real biological variation, whereas levels of the unloader WAPL and other cohesin accessories vary across cell types (Hill et al. 2020; Kiefer et al. 2023). Similarly, CTCF site occupancy depends on CTCF abundance, which has also been reported to vary roughly twofold between U20S and mESC cell lines (Cattoglio et al. 2019). Due to this complexity, we parameterized and compared simulations with mESC data in this project. As biophysical data become available for a greater number of cell types, future models can be parameterized to make cell type–specific predictions.

Despite considering a simplified scenario, where all sites had the same bound and unbound times, our estimated bound times are only slightly larger than those reported via the latest FRAP measurements (∼12 min vs. ∼10 min [Narducci and Hansen 2025]) and well within the broader range suggested by locus tracking experiments (5–30 min [Gabriele et al. 2022; Mach et al. 2022]). If we assumed a slightly lower occupancy (50% rather than 70%), our estimated bound times would increase (to ∼25 min). Incorporating additional details into future models of barrier dynamics could thus improve agreement with experimental measurements. First, site-specific CTCF bound times could enable a subpopulation of longer-lived CTCFs to provide most of the barrier functionality, yet be minimally reflected by current SPT or FRAP assays. Support for this comes from reports of stably bound CTCF populations (52.7 min in resting and 27.1 min in activated B cells [Kieffer-Kwon et al. 2017], 16.7 min in a human fibroblast cell line [Agarwal et al. 2017]) as well as persistent protection of CTCF motifs after long MNase digestions (20 min, [Ramani et al. 2019]). Second, CTCF barrier activity involves a more complex mechanism than simply blocking extruders (Hansen 2020), perhaps via protection from WAPL (Li et al. 2020; Wutz et al. 2020; Liu and Dekker 2022; Brunner et al. 2025), preventing association with NIPBL (Rhodes et al. 2017), interaction with PDS5 (Wutz et al. 2017; Nora et al. 2020), or via a tension-dependent mechanism (Davidson et al. 2023). Indeed, if CTCF can deactivate the cohesin motor (Hansen 2020), its effective barrier bound time would be longer than that inferred from its dynamics alone. Detailed models of how CTCF might deactivate cohesin thus represent a fruitful topic of future research. However, this may require extending existing models (e.g., Nomidis et al. 2022) of how cohesin translocates along the chromatin fiber and making them computationally efficient enough to simulate multiple megabases of chromatin.

In sum, our work highlights the ability of models to triangulate across multiple experimental modalities, revealing signatures of dynamic barriers evident even in the fixed-time snapshots provided by genomic data sets. Going forward, understanding the balance between barrier and extruder dynamics will sharpen our understanding of how cells harness loop extrusion for myriad biological functions.

## Methods

### Loop extrusion simulations

Our simulations of loop extrusion with dynamic barriers integrated a 1D lattice model for loop extrusion with 3D polymer simulations of chromatin. This requires specifying the following timescales:

CTCF site bound time: τ_b_; CTCF site unbound time: τ_u_; extruder lifetime: τ_E_; extruder step rate: Δτ_E_; lattice update timescale: δt_lattice_; polymer update timescale: δt_3D_. From CTCF site bound time (τ_b_) and unbound time (τ_u_), we additionally define occupancy: o = τ_b_/(τ_b_ + τ_u_).

In total, we performed simulations with 10 consecutive replicas of a 2.5-Mb region of chromatin. The 1D lattice model comprised 100,000 sites, each representing 250 bp. Extruders were randomly loaded every 1000 sites (close to 240 kb) (Gabriele et al. 2022). Extrusion complexes stochastically dissociated after an average time determined by τ_E_. While associated, each extruder leg moved independently and progressed with fixed stepping probabilities unless stalled by collisions with other extruders or with unidirectional CTCF barriers. When barriers became unbound, stalled extruder legs could resume extrusion. A set of 32 randomly generated CTCF barriers were placed in each replica, yielding an approximate CTCF spacing of 75 kb (based on experimental estimates [Cattoglio et al. 2019]).

3D polymer simulations used polychrom (https://github.com/open2c/polychrom) and OpenMM (Eastman et al. 2017) to simulate 2.5 Mb of chromatin at 2.5-kb resolution per monomer. Loop extrusion was modeled by harmonic bonds linking extruder legs, with leg positions from the 1D lattice model. Molecular dynamics simulated time was calibrated using experimental chromatin diffusion as in Nuebler et al. (2018). Reference parameters for extruder lifetime and processivity were obtained from experimental measurements for cohesin (Hansen et al. 2017). Full simulation details, including the lattice setup, extruders update rules, barrier interaction definitions, polymer simulation parameters, and calibration procedures are available in Supplemental Methods.

### ChIP-seq and FRiP analysis

We computed in silico ChIP-seq by collecting the positions of extruder legs from a total of 10,000 instances of the 1D lattice simulations. We tabulated extruder leg counts per lattice site, averaged over all 10 replicas. From in silico ChIP-seq, we then computed the fraction of reads in peaks as the sum of extruder legs at barrier positions, relative to the total number of extruder legs.

To quantify experimental ChIP-seq data, we analogously calculated FRiP for cohesin ChIP-seq reads in CTCF peaks, using the fastaFRiP pipeline (https://github.com/Fudenberg-Research-Group/fastaFRiP), deployed with Snakemake (Mölder et al. 2021). Briefly, we aligned FASTQ data to the mm10 genome with Bowtie 2 (Langmead and Salzberg 2012) and called peaks with MACS2 (Zhang et al. 2008). Using the default narrowPeak width for CTCF peaks, we then counted the number of cohesin reads in CTCF peaks with deepTools (Ramírez et al. 2016) and divided by the total number of mapped reads to obtain experimental FRiP per data set.

### Hi-C analysis

We generated in silico Hi-C maps from polymer conformations using polychrom and converted them to cooler format for comparison with experimental data. Quantitative analysis of contact maps, including computation of dot and insulation scores, was performed using chromoscores (https://github.com/Fudenberg-Research-Group/chromoscores/), which first extracts snippets from simulated maps around barriers or pairs of barriers. Insulation and dot scores are then computed on average snippets from maps binned to 10-kb resolution. To compute the insulation score from an average snippet centered on the barrier, we used triangular regions (window size 50 kb) upstream, downstream, and spanning the barrier as a natural way to avoid influence from the first few diagonals (Falk et al. 2019). This choice weights contacts at each distance similarly for the within- and between-domain areas and results in a consistent score for either observed or observed/expected maps. To compute dot scores, we computed average snippets (window size 80 kb) centered around pairs of barriers as a function of genomic distance in 25 logarithmically spaced bins between 100 kb and 5000 kb. For convergent dot scores, we only used snippets around pairs of convergently oriented barriers. From the average snippets, dot scores as a function of distance were computed as the ratio of contacts in the center relative to four control regions. To obtain a single dot score, we computed the average across all genomic distances, weighted by the number of snippets at each genomic distance to account for variation in the number of snippets as a function of genomic distance.

We analyzed experimental Hi-C at the same 10-kb resolution as simulated data. We used Open2C tools cooler (Abdennur and Mirny 2020), pairtools (Open2C et al. 2024c), and distiller (https://zenodo.org/records/7309110) to process reads into binned Hi-C contact maps, and *cooltools* (Open2C et al. 2024a) to extract features from maps. To define experimental CTCF barrier positions for insulation and dot analysis, we obtained the overlap between positions of CTCF motifs based on their score from JASPAR (Rauluseviciute et al. 2024) and ChIP-seq peaks (from Justice et al. 2020) using bioframe (Open2C et al. 2024b). We then considered the strongest CTCF motif in any 10-kb bin (based on their score from MACS2), resulting in 29,986 positions across the genome. We computed dot and insulation scores from experimental Hi-C data entirely analogously to simulations by using *coolpup.py* to extract snippets and create pile-ups (Flyamer et al. 2020) for all pairs of barriers (using coolpup.py.pileup with features_format= ‘bed'). Dot calling in Supplemental Figure S5 was performed with Mustache (Roayaei Ardakany et al. 2020).

### Vermicelli analysis

Vermicelli score quantifies the spatial relationship between DNA and cohesin positions within a 3D space. We performed a 3D rasterization of these positions into a voxel grid and subsequently applied Gaussian smoothing to the grids. The score was then calculated as the Pearson's correlation between the smoothed voxel grids of monomers and extruder positions.

### Software availability

Code specifying simulations and for performing analysis is available at GitHub (https://github.com/Fudenberg-Research-Group/dynamic_extrusion_boundaries) and as Supplemental Code.

## Supplemental Material

Supplement 1

Supplement 2

Supplement 3
