# Supplementary figures and images for: Dynamic barriers modulate cohesin positioning and genome folding at fixed occupancy
Source: Genome Res. 2025 Aug;35(8):1745–57. doi: 10.1101/gr.280108.124 (PMC12315716; doi:10.1101/gr.280108.124)

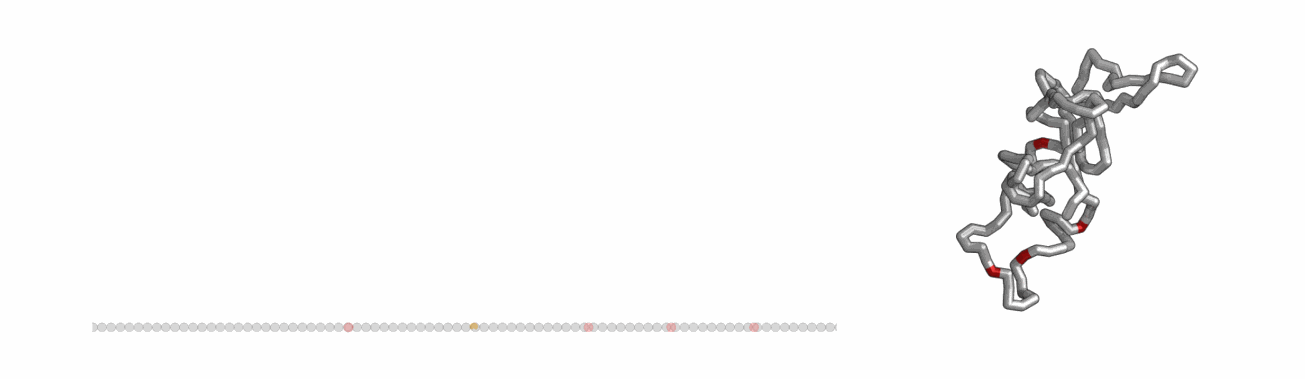

Supplement: Supplement 1 [file Supplemental_Movie_1.gif]
