# Supplementary material for: Dynamic barriers modulate cohesin positioning and genome folding at fixed occupancy
Source: Genome Res. 2025 Aug;35(8):1745–57. doi: 10.1101/gr.280108.124 (PMC12315716; doi:10.1101/gr.280108.124)
Supplement: Supplement 3 [file Supplemental_Material.pdf]

Table of Contents

Supplementary Table **2**
Table S1. Experimentally reported SPT and FRAP for CTCF and cohesin in mouse cells. 2
Supplementary Figures **3**
Figure S1 . Loop size depends on dynamic barrier parameters. 3
Figure S2. FRiP dependency on extruder parameters and CTCF clusters. 4
Figure S3. Frip for clusters of neighboring CTCF sites. 5
Figure S4. insulation strength of boundaries versus extruder lifetime. 6
Figure S5. Dot-detection-based methods display reference sample bias for dot enrichment scores 7
Figure S6. Dot scores depend on extruder lifetime. 9
Figure S7. Direct loops and stacked loops occur between convergent barriers at similar rates. 12
Supplemental Methods **13**
Loop extrusion and 3D polymer simulations 13
1D lattice model of loop extrusion 13
3D polymer simulations 14
Hi-C analysis 15
Analytical formula for loop size 16
Supplemental References **18**

Supplementary Table

paper	protein	condition	cell-type/cell-line	method	bound time, s
Hansen, 2017	CTCF	WT	mESC C59	SPT	61
Hansen, 2017	CTCF	WT	mESC C87	SPT	63
Hansen, 2017	CTCF	WT	mESC, C87	FRAP (11min 0.5hz)	247
Hansen, 2017	CTCF	WT	mESC, C87	FRAP (5min, 1hz)	183
Hansen, 2017	CTCF	WT	mESC, C59	FRAP (11min 0.5hz)	263
Hansen, 2017	CTCF	WT	mESC, C59	FRAP (5min, 1hz)	197
Hansen, 2020	CTCF	CTCF-RBRi	mESCC59	FRAP (11min 0.5hz)	220
Soochit, 2021	CTCF	WT	mESC	FRAP (5min, 0.2hz)	140
Soochit, 2021	CTCF	CTCF-del8	mESC	FRAP (5min, 0.2hz)	17
Soochit, 2021	CTCF	CTCF-del9	mESC	FRAP (5min, 0.2hz)	similar to WT
Soochit, 2021	CTCF	CTCF-del10	mESC	FRAP (5min, 0.2hz)	similar to WT
Soochit, 2021	CTCF	CTCF-del11	mESC	FRAP (5min, 0.2hz)	similar to WT
Narducci, 2025	CTCF	WT	mESC, clone A	FRAP (10.5min, 0.25hz)	700
Narducci, 2025	CTCF	WT	mESC, clone B	FRAP (10.5min, 0.25hz)	700
Narducci, 2025	CTCF	WT	mESC, clone D	FRAP (10.5min, 0.25hz)	600
Narducci, 2025	CTCF	WT	mESC, C87	FRAP (10.5min, 0.25hz)	700
Tedeschi, 2013	RAD21	WT	MEF	FRAP	1500
Hansen, 2017	RAD21	WT	mESC	FRAP	1320
Morales, 2020	RAD21	WT	MEFs	FRAP	1776

Table S1. Experimentally reported SPT and FRAP for CTCF and cohesin in mouse cells.

Note that tabulated cohesin observations were for the RAD21 subunit. Also note that instead of reporting numeric values, Soochit et al., 2021 reported the bound times for a few mutants as similar to WT.

Supplementary Figures

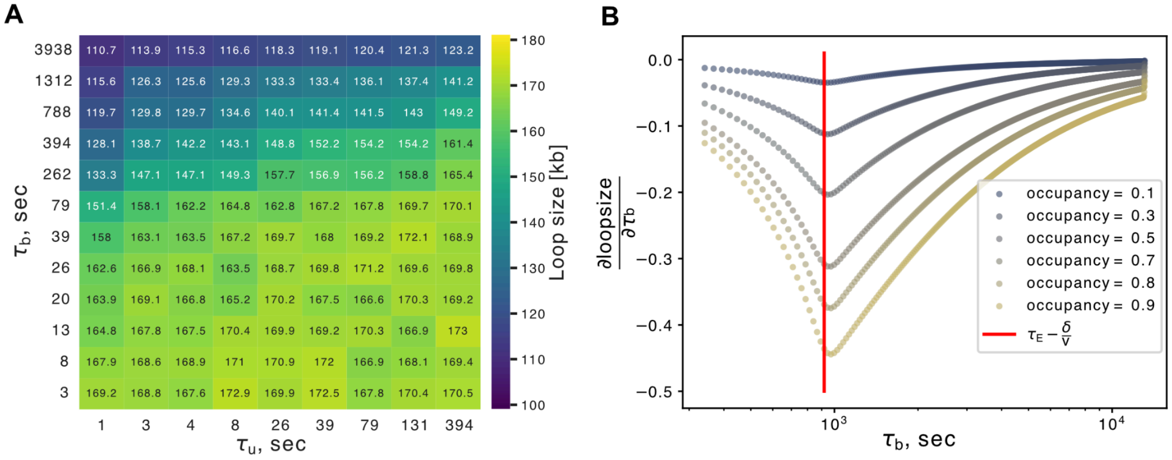

Figure S1 . Loop size depends on dynamic barrier parameters.

a. Heatmap of averaged loop size for simulations with multiple extruders and barriers, for the biologically-informed layout of CTCF positions used throughout the paper. This indicates similar behavior in loop size dependency on binding time, albeit lowered by collisions.

b. Derivative of analytical formulae for loop size as a function of $\boldsymbol{\tau}_b$ for various occupancies (ranging from 0.1 to 0.9), showing a minima at $\boldsymbol{\tau}_E$ - δ/v (vertical red line).

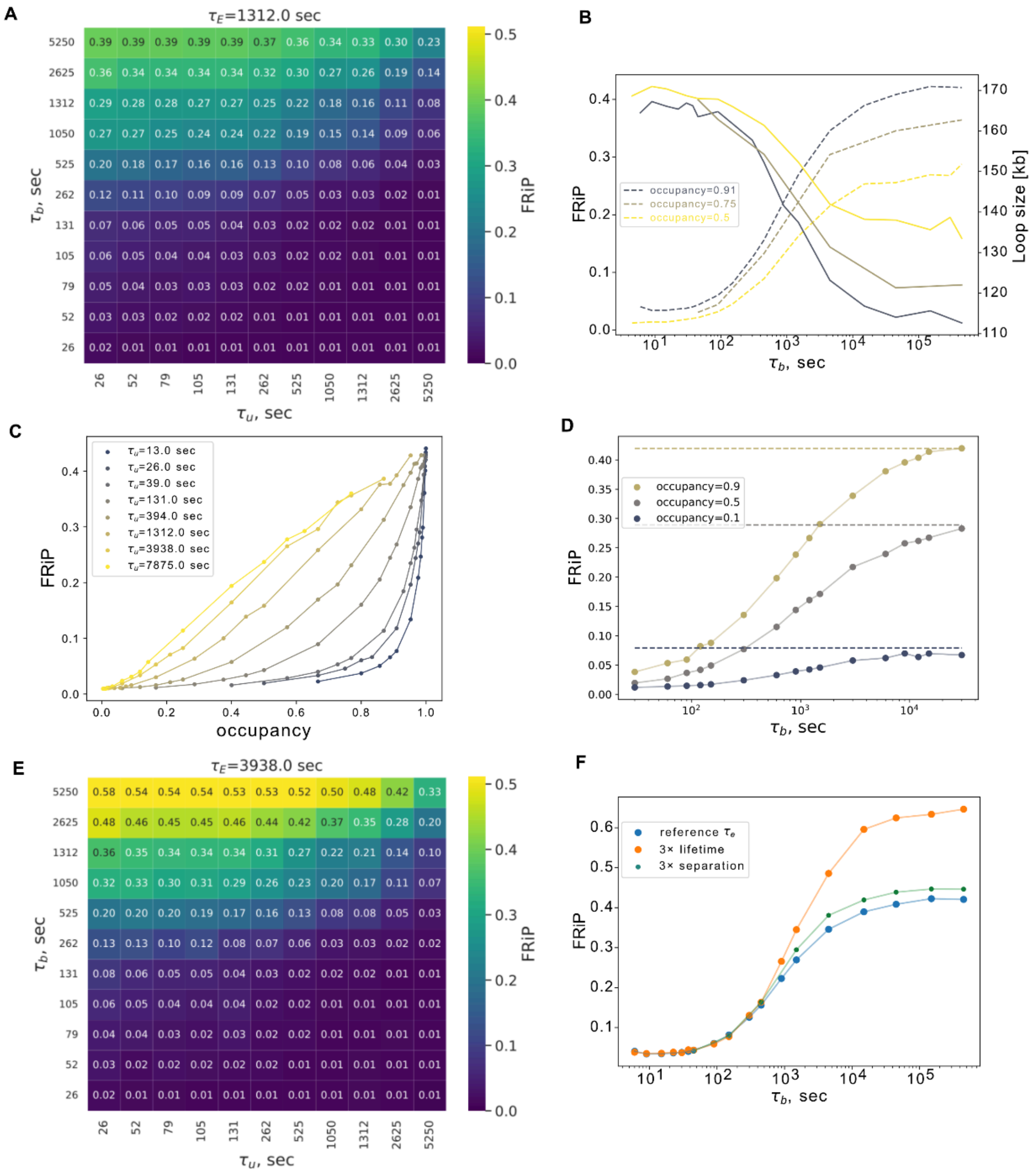

Figure S2. FRiP dependency on extruder parameters and CTCF clusters.

a. Heatmaps of FRiP for $\boldsymbol{\tau}_b$ vs $\boldsymbol{\tau}_u$. Note the strong dependence on barrier bound time (y-axis).

b. Inverse relationship between FRiP (dashed lines) and loop size (solid lines) as a function of barrier bound time, for multiple occupancies (shown with matching color for the two quantities).

c. FRiP as a function of occupancy. FRiP increases linearly with occupancy at high unbound times ($\boldsymbol{\tau}_u$) but increases sub-linearly when unbound time is similar to or below extruder lifetime ($\boldsymbol{\tau}_u <= \boldsymbol{\tau}_E$, $\boldsymbol{\tau}_E$ =1312 sec).

d. FRiP approaches its value for static barriers (dashed lines) at large $\boldsymbol{\tau}_b$

e. heatmap of FRiP for increased extruder lifetime, as a function of bound and unbound time**.**

f. FRiP vs $\boldsymbol{\tau}_b$ for reference simulations versus those with increased lifetime or separation. While three-fold increased lifetime had strong effect at large $\boldsymbol{\tau}_b$, three-fold increased separation had a weak effect.

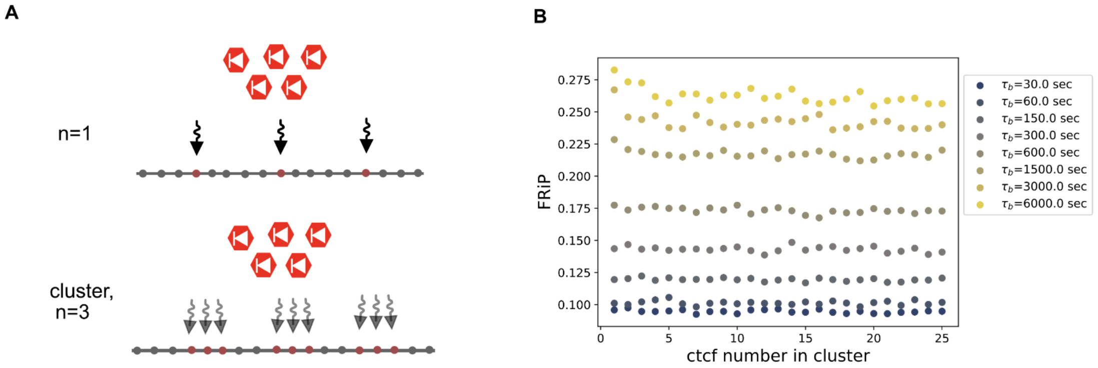

Figure S3. Frip for clusters of neighboring CTCF sites.

a. Illustration of clusters of CTCF sites with a fixed average number of bound CTCFs at 50% occupancy. In this scenario each site has an increased unbound time ($n \times \boldsymbol{\tau}_u$) and an occupancy 0.5/n.

b. FRiP as a function of CTCF number per cluster. To compute FRiP for clusters, we consider a fixed window (width=27, from one site upstream to one site downstream of the maximal cluster size considered) around the cluster of sites (in contrast to width=3, or +/-1 site, around each CTCF site for FRiP computed elsewhere).

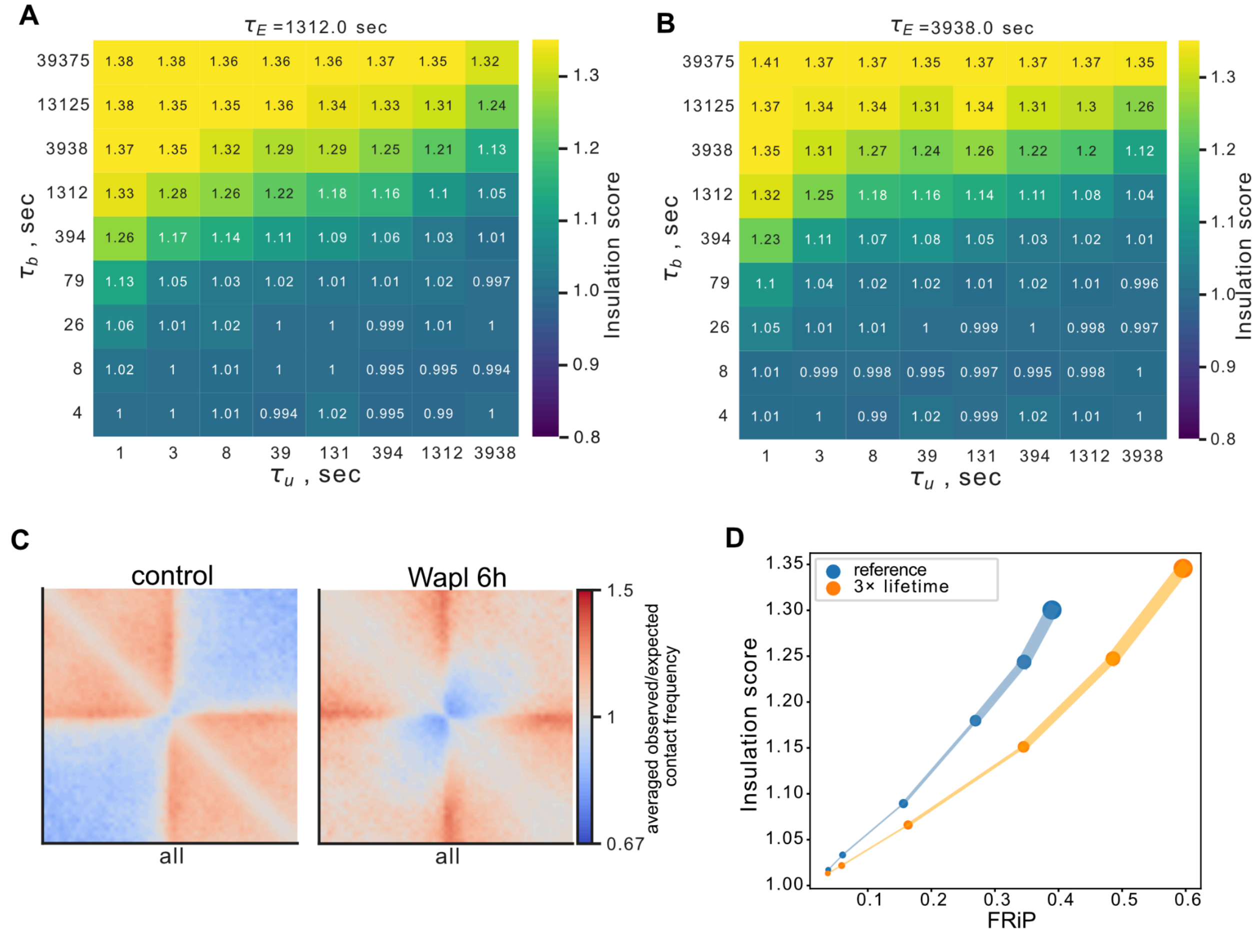

Figure S4. insulation strength of boundaries versus extruder lifetime.

a. Heatmap of insulation score as a function of barrier lifetime and unbound time.

b. Heatmap of insulation score for extruders with 3-times longer lifetimes than reference simulations.

c. Average contact map snippets around CTCF sites for Liu et al., 2021 control and dWAPL datasets (N. Q. Liu et al. 2021).

d. Scatterplot of insulation score versus FRiP for different barrier bound times, shown by point size. At a fixed extruder lifetime, insulation is correlated with FRiP. However, across extruder lifetimes (blue versus orange points), these scores were anti-correlated.

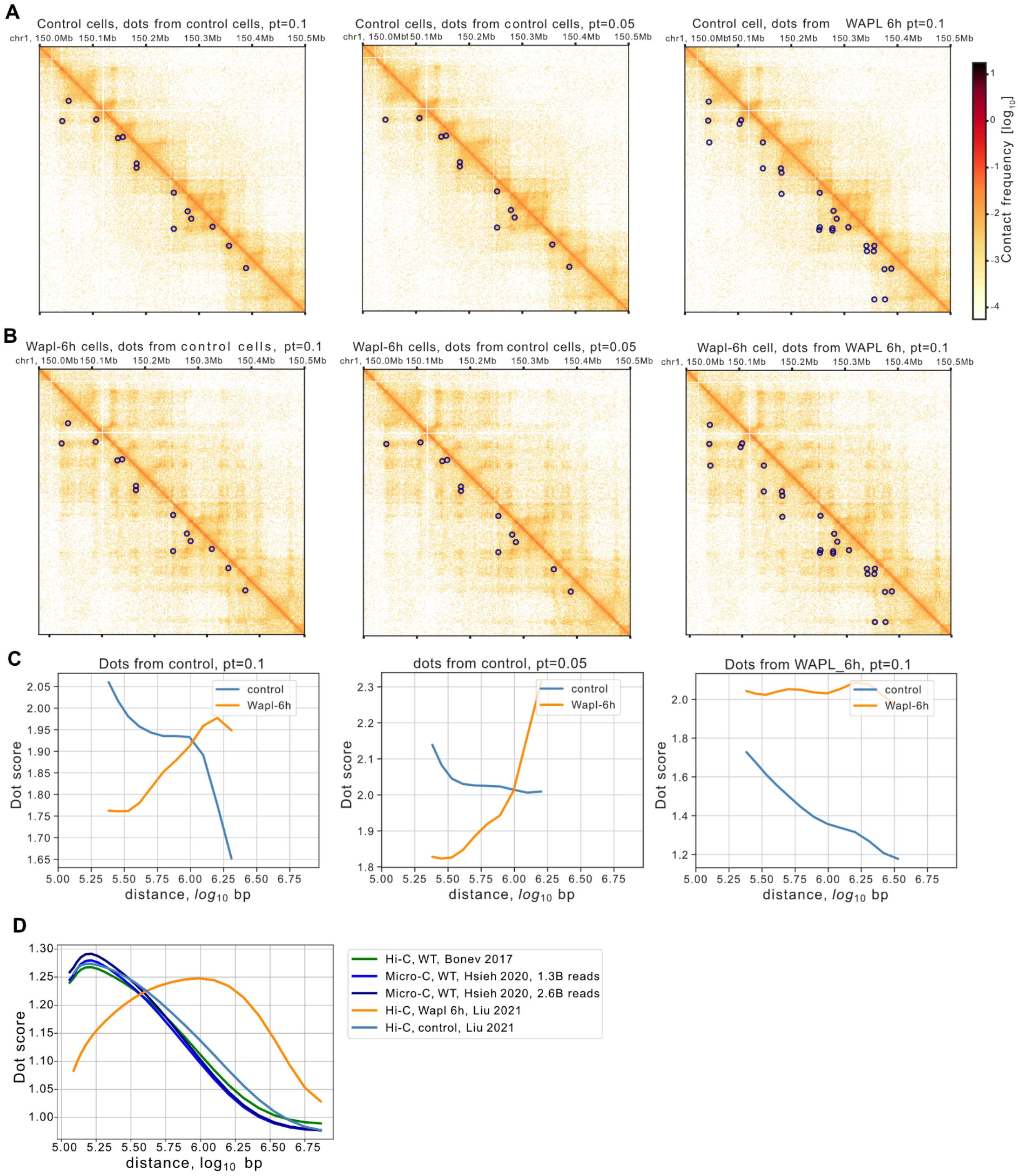

Figure S5. Dot-detection-based methods display reference sample bias for dot enrichment scores

a. Hi-C maps showing how detected dots depend on P-value thresholds and perturbation status. *Left:* Dots detected in control cells with a P-value threshold of 0.1, overlaid on a Hi-C map of control cells (i.e.

WAPL-0h). *Middle:* Same as left but with a P-value threshold of 0.05. *Right:* Dots detected in Wapl-depleted cells with a P-value threshold of 0.1, overlaid on control cell Hi-C maps. Dots were called using Mustache software (Roayaei Ardakany et al. 2020). Hi-C for control and WAPL-depleted cells from Liu et al., 2021.

b. Same as panel **a**, but the same three sets of called dots are overlaid on Hi-C maps of Wapl-depleted cells.

c. Dot scores versus distance as a function of genomic distance for the same three sets of called dots, filtered by overlapping convergent motifs. These plots illustrate how P-value threshold and cohesin perturbations influence computed dot scores. In particular, these plots illustrate a reference sample bias: the control lines are consistently higher on the set of dots called in control, but lower on the set of dots called in dWAPL. Also note the more stringent P-value threshold leads to higher scores. Also note that a dot calling approach leads to higher dot scores versus distance than our barrier-based analysis.

d. Convergent dot score as a function of genomic distance for Hi-C and Micro-C mESC datasets, using a barrier-based analysis, indicating a similar trend and comparable magnitude, as in **Fig. 5B**.

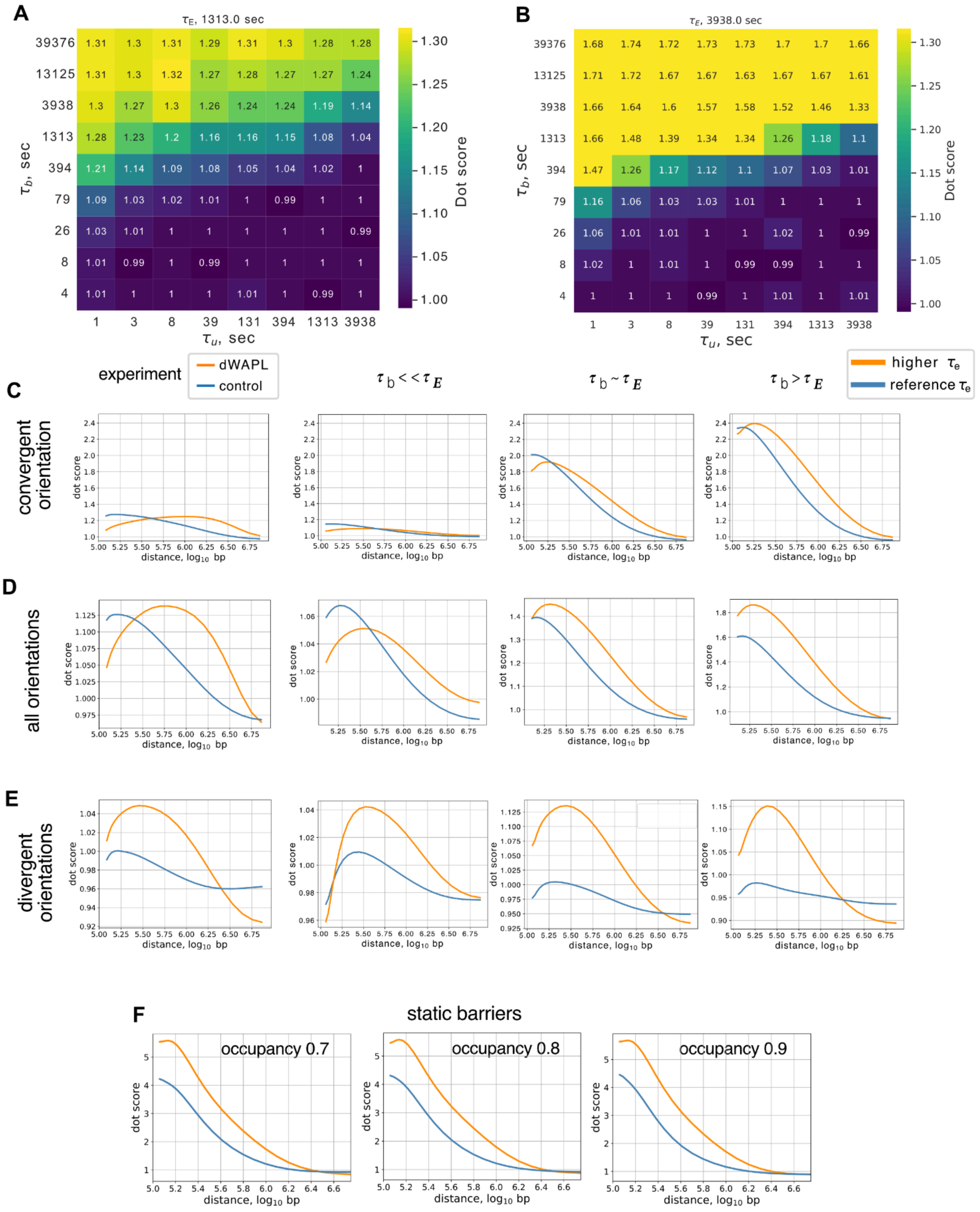

Figure S6. Dot scores depend on extruder lifetime.

a. Heatmap of dot score for barrier bound versus unbound times.

b. Similar heatmap at 3x increased extruder lifetime.

c. Comparison of the magnitude of convergent dot scores versus genomic distance for experimental data and simulations at various $\boldsymbol{\tau}_b$ regimes, supporting the biological relevance of the $\boldsymbol{\tau}_b < \boldsymbol{\tau}_E$ regime. Note the shared y-axis across all plots.

d. Overall dot score (i.e., averaged across all barrier orientations) as a function of distance, for extruders with either reference (blue) or higher (orange) lifetimes at three CTCF bound times. Only simulations with $\boldsymbol{\tau}_b<\boldsymbol{\tau}_E$ display similar behavior to experiments, where the higher-lifetime curve starts lower and has a peak after the wild-type curve.

e. Dot score for divergently oriented barriers (<>) as a function of distance. Note that these divergent scores are generally lower than for convergent barriers. Also note that experimental data for WAPL depletion has higher divergent dot scores than the control, even at relatively short genomic distances. In simulations, where barriers are strictly uni-directional, enriched divergent dot scores (i.e., greater than one) can arise via stacking of extruders against a CTCF-stalled extruder.

f. Dot score versus distance in simulations with static barriers (shown for three occupancies) is always higher when extruder lifetime is increased. This behavior is similar to that of dynamic barriers with large $\boldsymbol{\tau}_b$ (i.e., $\boldsymbol{\tau}_b>>\boldsymbol{\tau}_E$), yet differs from what is observed in experimental data.

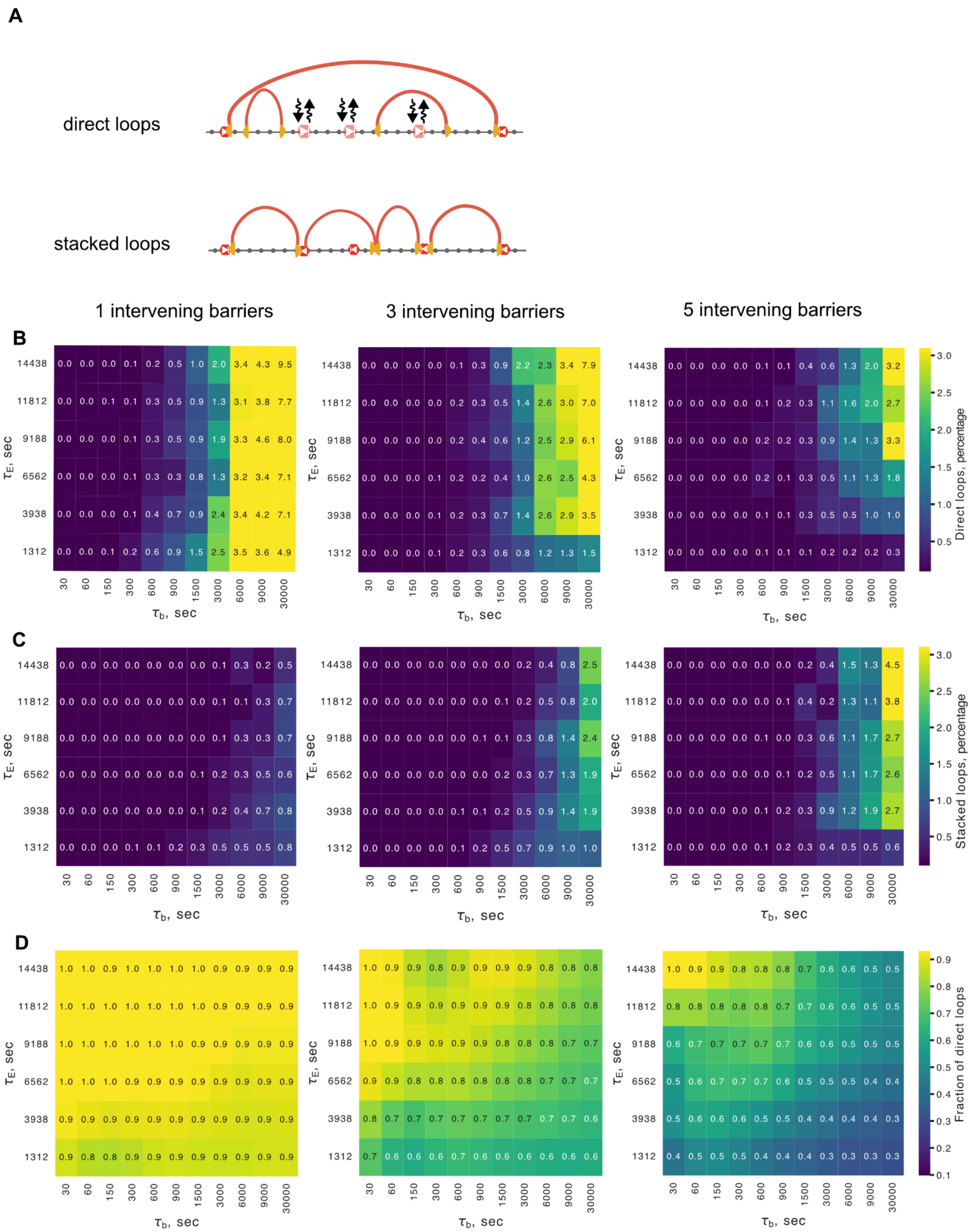

A
direct loops
stacked loops
1 intervening barriers
3 intervening barriers
5 intervening barriers
B
C
D
τ_E, sec
τ_b, sec
Direct loops, percentage
Stacked loops, percentage
Fraction of direct loops

Figure S7. Direct loops and stacked loops occur between convergent barriers at similar rates.

a. Illustration of distant convergent barriers separated by three intervening barriers connected with either (*top*) a direct loop via bypass or (*bottom*) versus stacked loops between distant convergent barriers.

b. Heatmaps of direct loop frequency between convergent barriers, for 1 intervening barrier (left), 2 intervening barriers (middle) and 3 intervening barriers (right).

c. Heatmaps of stacked loop frequency at the same barrier separations.

d. Fraction of direct loops relative to the total loops between the convergent barriers at the same separations. The fraction of direct loops between convergent barriers decreases with increasing number of intervening barriers, as well as higher barrier lifetimes; the fraction of stacked loops does the opposite.

Supplemental Methods

Loop extrusion and 3D polymer simulations

1D lattice model of loop extrusion

For a 2.5Mb region of chromatin, we simulated extrusion using a one-dimensional lattice with 10,000 sites, each representing 250 bp. At each time step, extruders that were not stalled at barriers took outward steps with 10% probability. Together this implementation enabled fixed-timestep simulations with rapid CTCF exchange (CTCF bound time up to 30 times lower than cohesin), at close to nucleosome resolution. Loop extruders are loaded randomly on the lattice, with a number depending on the separation parameter. For our simulated reference condition (reflecting wild type mESCs) this was 250kb (1000 sites), close to that in previous simulations (240kb, (Gabriele et al. 2022)). A loop extruder occupies two lattice sites, one for its right and one for its left leg. Loop enlargement is modeled by re-assigning legs to new lattice sites, where an extruder at (i, j) gets reassigned to (i-1, j+1). Legs of different cohesins cannot bypass each other when they meet. Extruders cannot translocate off the lattice from either the first or last lattice site. We considered CTCF sites as purely unidirectional barriers with time-varying occupancy: only cohesins whose extrusion direction clashes with the barrier orientation are stalled, and only when the barrier is occupied. We also considered each leg as operating independently: if a leg is stalled at an occupied barrier, it no longer moves until that barrier unbinds the lattice; if the other leg is unimpeded it can still extrude outwards independently. Eventually, extruders dissociate from the lattice with a fixed rate determined by the extruder lifetime, which also governs processivity (the average extruded distance without collision), and are loaded randomly at a new position.

To speed up averaging, we used 10 consecutive replicas of the same layout of 10,000 sites, giving a full lattice of 100,000 sites. Each replica had the same positions of 32 randomly-generated barrier positions, with 16 right-stalling barriers at: [314, 579, 1195, 3717, 3772, 3921, 4451, 5193, 5723, 6302, 6574, 6779, 7000, 9232, 9310, 9861], and 16 left-stalling barriers at: [495, 865, 1404, 2164, 3143, 3615, 3971, 4069, 4480, 4938, 5300, 5587, 6401, 7725, 8764, 9619]. On average, this corresponds to 75 kb (300 sites) between barriers, approximating experimental estimates (74.1 kb from ~217,000 CTCFs per nucleus, 50% bound, and the average genomic content of roughly 3×2.7 Gbp (Cattoglio et al. 2019)).

We performed 10,000 update steps to generate arrays of extruder positions over time. With reference simulation parameters, reflecting an experimental wild type scenario, extruders had a processivity of 1000 lattice steps (250 kb) and separation of 1000 sites. We initialized simulations with barrier occupancy states chosen proportional to their barrier binding time relative to the sum of their binding and unbinding time.

3D polymer simulations

We modeled 2.5 Mb of chromatin as a 50 nm fiber, where each monomer represents 2.5 kb (i.e., 10 1D lattice sites). We chose this resolution for the 3D simulations to reduce computational cost yet still be below a typical experimental Hi-C analysis resolution of 10kb. We used polychrom (GitHub - open2c/polychrom) to implement polymer models, which harnesses OpenMM (Eastman et al. 2017) to generate large ensembles of conformations. We introduced an extra harmonic bond between the two monomers connected by each pair of extruder arms (i, j). When the loop extrusion factor (LEF) advances,

this bond is removed and replaced by a new bond between (i-1, j+1). We observe Rouse dynamics for our wild-type scenario and calibrated simulation timescales with experimental timescales by aligning Mean Square Displacement (MSD) curves. Assuming a diffusion coefficient $D = 0.01\ \mu m^2/s^{0.5}$(as in (Nuebler et al. 2018)) with the monomer size used in these simulations (50 nm) each simulated time step in 3D represents δt_{3d} = 0.015 sec. We employed 175 3D steps between each 1D lattice update, so a lattice timestep $\delta t_{lattice}$=17.5 δt_{3d} amounted to about 2.6 sec of physical time, yielding a stepping rate of 2×250bp / 2.6sec = 190bp/sec. Thus, the reference extruder processivity of 500 lattice steps for each leg translated to an extruder lifetime of 21.8 min, consistent with experimental measurements for cohesin (Hansen et al., 2017). The timescales of barriers (binding and unbinding times, $\boldsymbol{\tau}_b$ and $\boldsymbol{\tau}_u$) are then calibrated compared to the extruder lifetime. Other parameters in our 3D simulations, including pairwise interaction potential, persistence length, and Langevin integration rate, were the same as those in(Nuebler et al. 2018)

Hi-C analysis

For *in silico* Hi-C, we generated a total of 10,000 conformations from polymer simulations. From these conformations, we created Hi-C maps at a resolution of one monomer size (2.5 kb) using the default polychrom capture radius (2.3 monomers) to record pairs of monomers in contact, approximating literature values (McCord et al. 2020). To improve statistical accuracy, we calculated contacts within subchains of 3,000 monomers for conformations after the system reached a steady state, and averaged the results across subchains and replicates. For a consistent analysis between simulated and experimental data, we converted simulated contact maps to cooler (Abdennur and Mirny 2020) format using polykit (https://github.com/open2c/polykit).

Analytical formula for loop size

Considering extrusion behavior across the three regimes described in the main text, we derive analytical equations for the loop size (L) in terms of extruder lifetime (τ_E), extrusion rate (v), barrier bound time (τ_b), unbound bound time (τ_u), and the distance between barriers (δ). Importantly this derivation neglects the impact of extruder-extruder collisions, which generally lower loop sizes. To write equations for the second and third regime succinctly, it is helpful to introduce probabilities of the barrier being occupied (o) or unoccupied ($1 - o$). Occupancy is simply proportional to the ratio of bound times and unbound times: $o = \frac{\tau_b}{\tau_b + \tau_u}$.

1. First regime ($\tau_E < \delta/v$). The extruder enlarges the loop without impediment and its size is simply the extrusion rate (v) multiplied by extruder lifetime : $L = v\,\tau_E$
2. Second regime ($\delta/v < \tau_E < \delta/v + \tau_b$). The extruder reaches the barriers, separated by δ, after a duration δ/v. Its remaining extruder lifetime ($\delta\tau = \tau_E - \frac{\delta}{v}$) may be spent either: (i) at the barrier if it is occupied, or (ii) continuing to extrude if the barrier is not occupied (proportional to $1 - o$): $L = \delta + (1 - o)\,v\,\delta\tau$.
3. Third regime ($\tau_E > \delta/v + \tau_b$). When the extruder binding time exceeds that of the barrier plus the time required to reach the barrier, extrusion can also proceed after being temporarily blocked by the barrier. After reaching the barrier, the extruder thus: (i) continue unimpeded if the barrier is unoccupied (proportional to $1 - o$); or (ii) become blocked at the barrier (proportional to o), yet proceed when the barrier unbinds (after its bound time τ_b). The resulting equation is:

$$L = \delta + (1 - o)\,v\,\delta\tau + o\,v\,(\delta\tau - \tau_b)$$

Substituting for occupancy yields:

$L = v\,\tau_E$, $\qquad\qquad$ $\tau_E < \delta/v$

$$L = \delta + \frac{\tau_u}{\tau_b+\tau_u} v (\tau_E - \frac{\delta}{v}), \qquad \delta/v < \tau_E < \delta/v + \tau_b$$

$$L = \delta + \frac{\tau_u}{\tau_b+\tau_u} v (\tau_E - \frac{\delta}{v}) + \frac{\tau_b}{\tau_b+\tau_u} v (\tau_E - \frac{\delta}{v} - \tau_b), \qquad \tau_E > \delta/v + \tau_b$$

As a barrier may be bound before the arrival of the extruder, we can obtain a more accurate expression for loop size by accounting for the fraction of bound time, f (0<= f <=1), that had elapsed prior to the arrival of the extruder, and substitute $f\tau_b$ for τ_b:

$$L = v\tau_E, \qquad \tau_E < \delta/v$$

$$L = \delta + \frac{\tau_u}{\tau_b+\tau_u} v (\tau_E - \frac{\delta}{v}), \qquad \delta/v < \tau_E < \delta/v + f\tau_b$$

$$L = \delta + \frac{\tau_u}{\tau_b+\tau_u} v (\tau_E - \frac{\delta}{v}) + \frac{\tau_b}{\tau_b+\tau_u} v (\tau_E - \frac{\delta}{v} - f\tau_b), \qquad \tau_E > \delta/v + f\tau_b$$

A final expression for loop size can then be obtained by integrating over f:

$$L = v\tau_E \qquad \tau_E < \delta/v$$

$$L = \delta + \frac{\tau_u}{\tau_b+\tau_u} v (\tau_E - \frac{\delta}{v}) + \frac{\tau_b}{\tau_b+\tau_u} v \frac{(\tau_E - \frac{\delta}{v})^2}{2\tau_b} \qquad \delta/v < \tau_E < \delta/v + \tau_b$$

$$L = \delta + \frac{\tau_u}{\tau_b+\tau_u} v (\tau_E - \frac{\delta}{v}) + \frac{\tau_b}{\tau_b+\tau_u} v (\tau_E - \frac{\delta}{v} - \frac{\tau_b}{2}) \qquad \delta/v + \tau_b < \tau_E$$

Supplemental References

Abdennur N, Mirny LA. 2020. Cooler: scalable storage for Hi-C data and other genomically labeled arrays. *Bioinformatics* **36**: 311–316.

Cattoglio C, Pustova I, Walther N, Ho JJ, Hantsche-Grininger M, Inouye CJ, Hossain MJ, Dailey GM, Ellenberg J, Darzacq X, et al. 2019. Determining cellular CTCF and cohesin abundances to constrain 3D genome models eds. D.J. Sherratt, K. Struhl, and D.J. Sherratt. *eLife* **8**: e40164.

Eastman P, Swails J, Chodera JD, McGibbon RT, Zhao Y, Beauchamp KA, Wang L-P, Simmonett AC, Harrigan MP, Stern CD, et al. 2017. OpenMM 7: Rapid development of high performance algorithms for molecular dynamics. *PLOS Comput Biol* **13**: e1005659.

Gabriele M, Brandão HB, Grosse-Holz S, Jha A, Dailey GM, Cattoglio C, Hsieh T-HS, Mirny L, Zechner C, Hansen AS. 2022. Dynamics of CTCF- and cohesin-mediated chromatin looping revealed by live-cell imaging. *Science* **376**: 496–501.

Liu NQ, Maresca M, van den Brand T, Braccioli L, Schijns MMGA, Teunissen H, Bruneau BG, Nora EP, de Wit E. 2021. WAPL maintains a cohesin loading cycle to preserve cell-type-specific distal gene regulation. *Nat Genet* **53**: 100–109.

McCord RP, Kaplan N, Giorgetti L. 2020. Chromosome Conformation Capture and Beyond: Toward an Integrative View of Chromosome Structure and Function. *Mol Cell* **77**: 688–708.

Nuebler J, Fudenberg G, Imakaev M, Abdennur N, Mirny LA. 2018. Chromatin organization by an interplay of loop extrusion and compartmental segregation. *Proc Natl Acad Sci* **115**: E6697–E6706.

Roayaei Ardakany A, Gezer HT, Lonardi S, Ay F. 2020. Mustache: multi-scale detection of chromatin loops from Hi-C and Micro-C maps using scale-space representation. *Genome Biol* **21**: 256.
